# Supplementary material for: Capture Hi-C identifies putative target genes at 33 breast cancer risk loci
Source: Nat Commun. 2018 Mar 12;9:1028. doi: 10.1038/s41467-018-03411-9 (PMC5847529; doi:10.1038/s41467-018-03411-9)
Supplement: Supplementary file 1 — Supplementary Information [file 41467_2018_3411_MOESM1_ESM.pdf]

Supplementary Information

Baxter et al.

Supplementary Figures 1-9

Supplementary Tables 1-3

Supplementary Figure 1: Schematic of Capture Hi-C method for identification of target genes at GWAS risk loci.

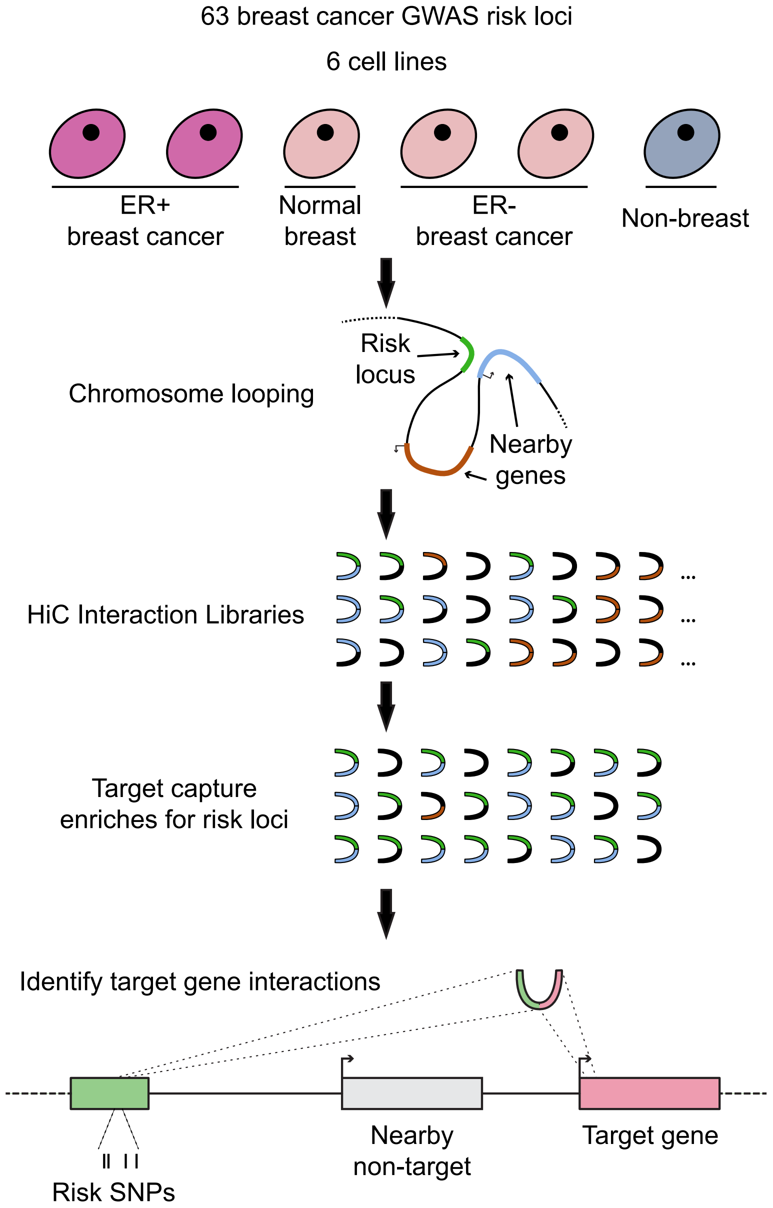

Legend: Hi-C libraries generated in four breast cancer, one normal breast epithelial and one lymphoblastoid cell line were subjected to target enrichment using arrays that captured linkage disequilibrium blocks corresponding to 63 breast cancer risk loci. Putative target genes defined as genes mapping within, or *in cis,* to a captured region for which the transcription start site mapped to an interacting fragment were identified.

Supplementary Figure 2: Histogram showing the number of interaction peaks per locus across 51 informative loci in six cell lines.

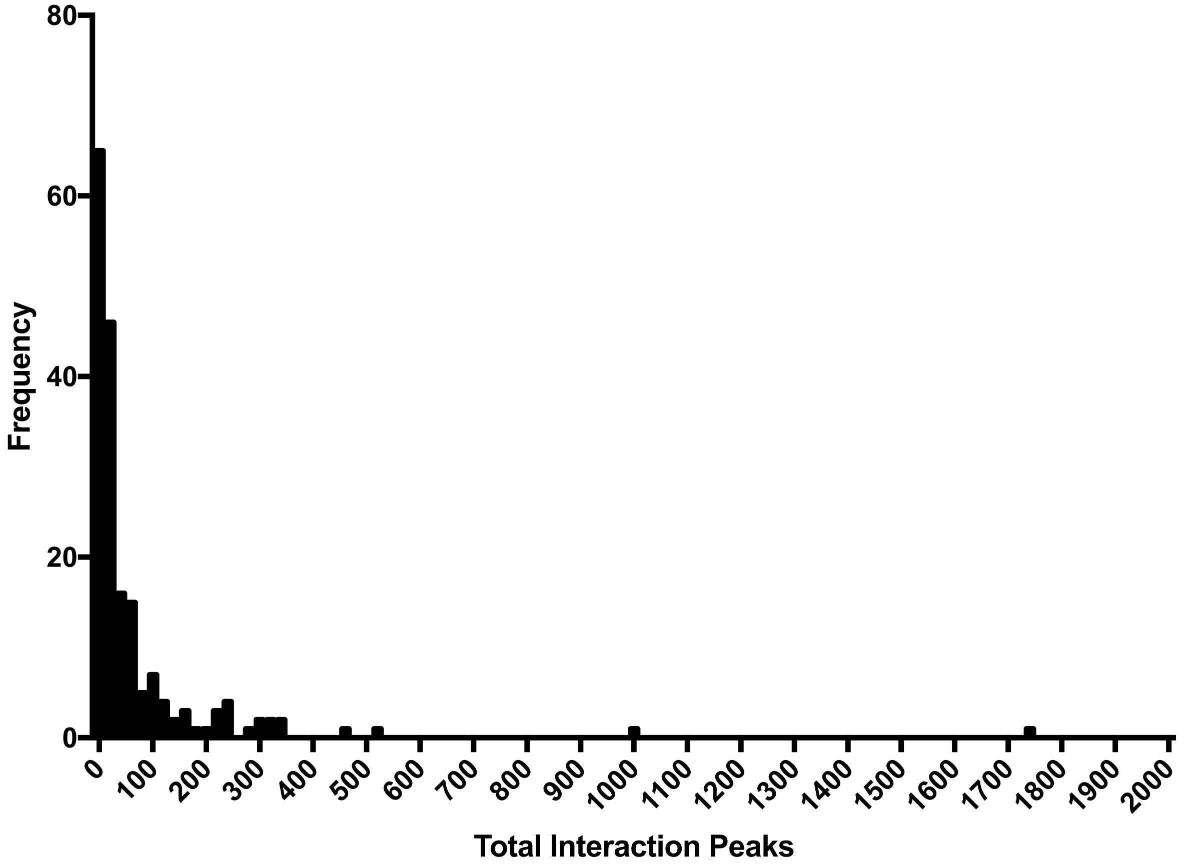

Legend: The number of interaction peaks per locus at 51 loci that had at least one interaction peak in at least one cell line is shown as a histogram. Loci with zero interaction peaks are excluded (N=18 (T-47D), N=15 (ZR-75-1), N=11 (Bre80), N=31 (BT-20), N=25 (MDA-MB-231) and N=29 (GM06990)). Data from all six cell lines are combined; two outliers (1,744 at 8q21.11 (rs2943559) in ZR-75-1 and 1,007 at 8q24.21 (rs13281615) in T-47D) are apparent.

Supplementary Figure 3: Long-range (>2 Mb) interaction peaks at 3p26.1, 4q24, 8q24.21 and 11q13.1 in Bre80 (Br), MDA-MB-231 (M), ZR-75-1 (Z) and GM06990 (G) cell lines aligned with topologically associated domains (TADs) in human mammary epithelial cells (HMEC).

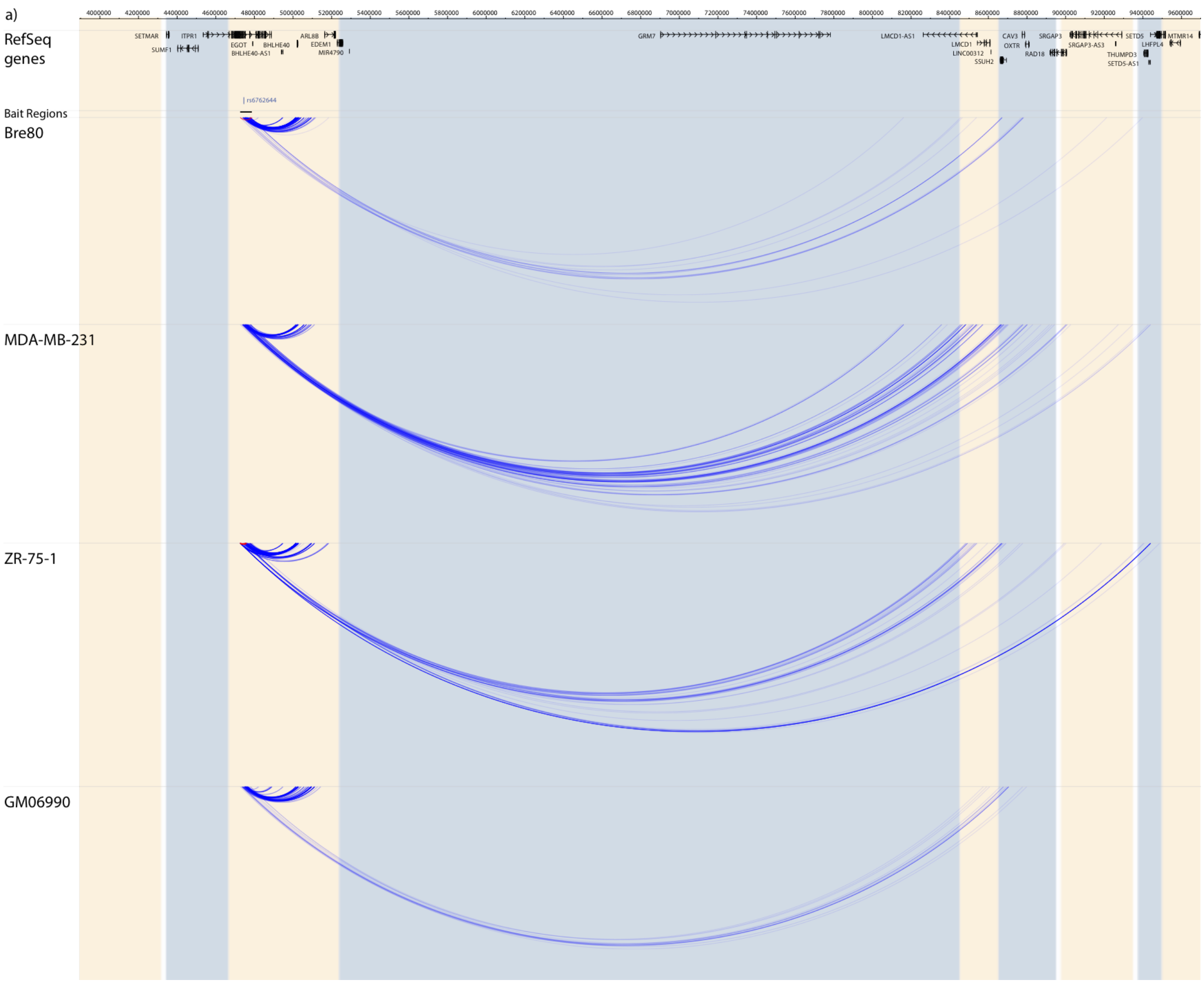

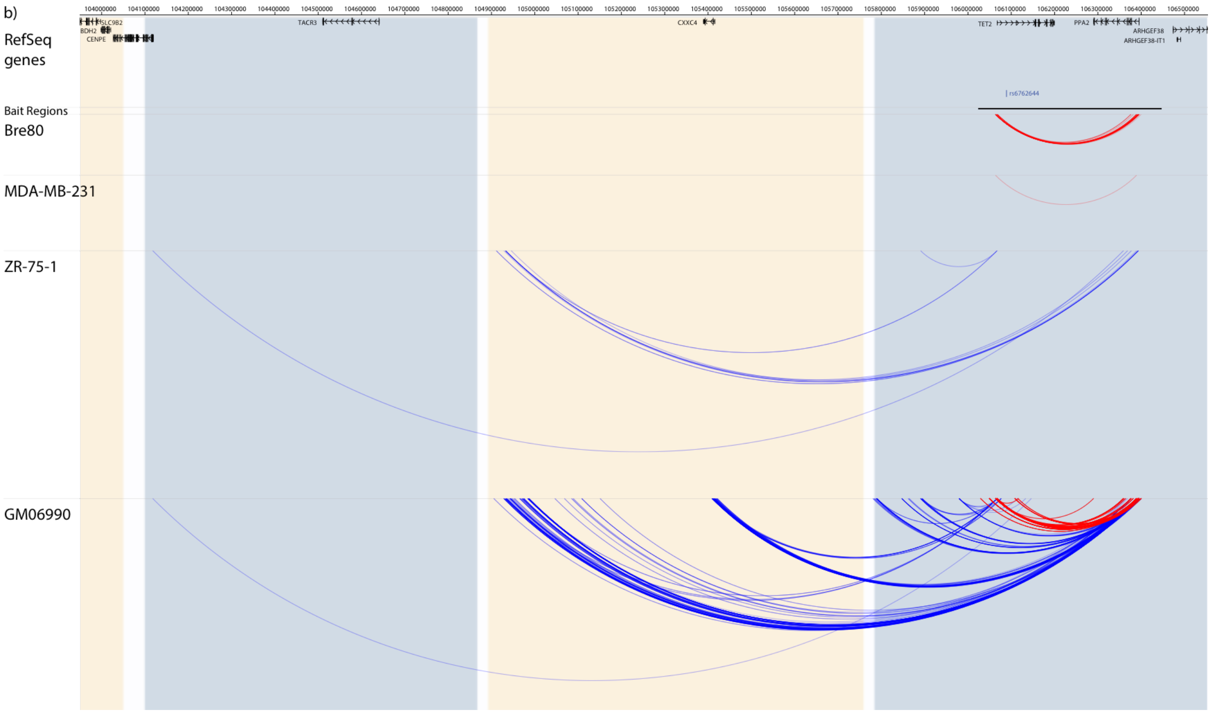
b)
RefSeq genes
Bait Regions
Bre80
MDA-MB-231
ZR-75-1
GM06990
104000000
104100000
104200000
104300000
104400000
104500000
104600000
104700000
104800000
104900000
105000000
105100000
105200000
105300000
105400000
105500000
105600000
105700000
105800000
105900000
106000000
106100000
106200000
106300000
106400000
106500000
SLC9B2
BDH2
CENPE
TACR3
CXXC4
TET2
PPA2
ARHGEF38
ARHGEF38-IT1
rs6762644

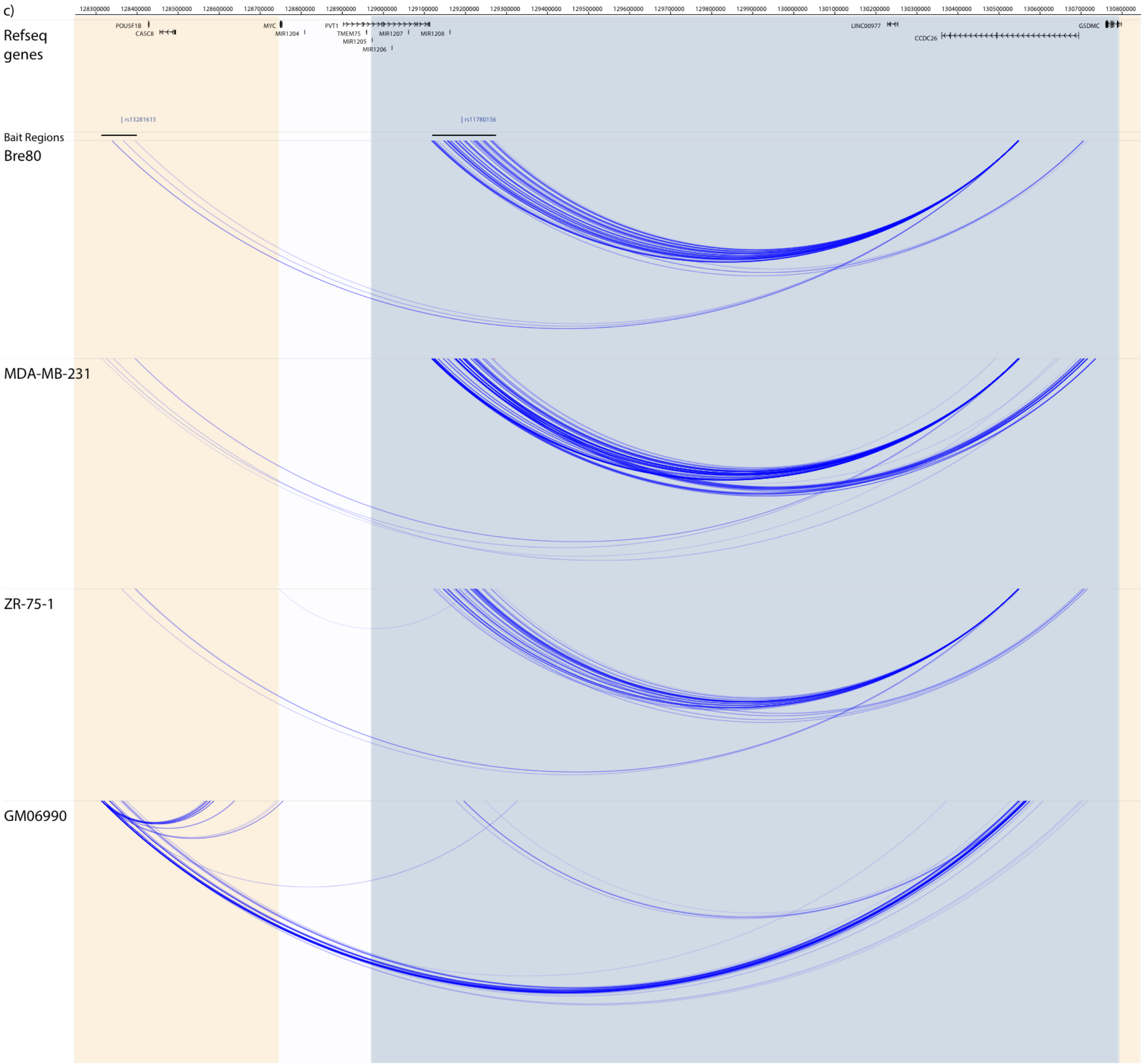

c)
128300000
128400000
128500000
128600000
128700000
128800000
128900000
129000000
129100000
129200000
129300000
129400000
129500000
129600000
129700000
129800000
129900000
130000000
130100000
130200000
130300000
130400000
130500000
130600000
130700000
130800000
Refseq genes
POU5F1B
CASC8
MYC
MIR1204
PVT1
TMEM75
MIR1205
MIR1206
MIR1207
MIR1208
LINC00977
CCDC26
GSDMC
rs13281615
rs11780156
Bait Regions
Bre80
MDA-MB-231
ZR-75-1
GM06990

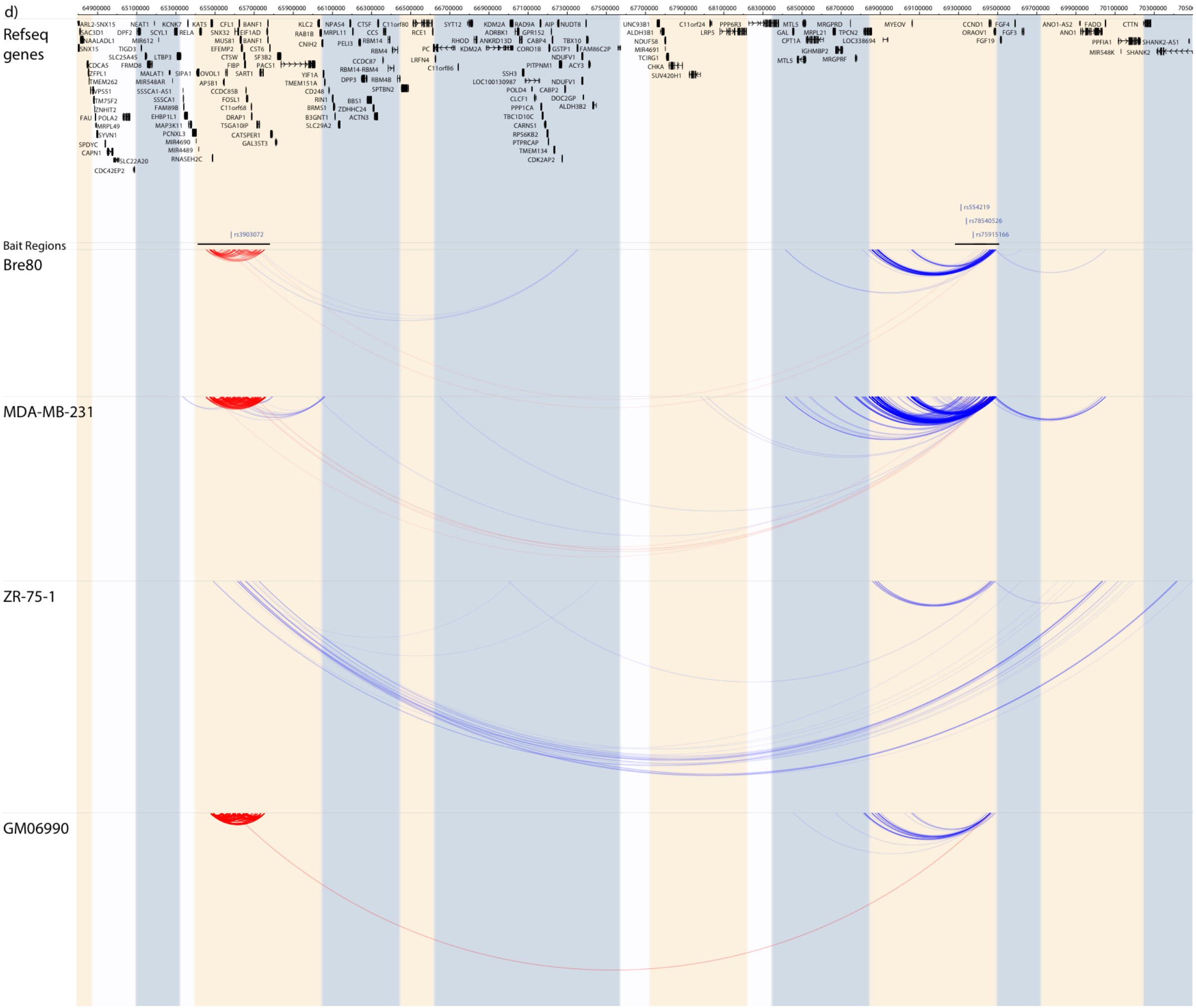

Legend: Interaction peaks (shown in a looping format) are aligned with TADs (shown as alternating blue and beige blocks). TAD boundaries were assigned using data generated in HMECs[22]. Interaction peaks between two captured fragments are red, interaction peaks between one captured fragment and one non-captured fragment are blue. Intensity of individual interactions are proportional to $-\log_2(P_{FDR})$. Capture regions are shown as black bars; data are aligned with genomic coordinates (hg19) and RefSeq genes. The subset of cell lines in which interaction peaks with specific target genes are observed are listed after the gene based on the subset of cell lines shown (full details are in Supplementary Table 5) (a) 3p26.1-rs6762644 targets *CAV3* (all), *LMCD1* (Z, M), *c3orf32* (Z, M, G), *RAD18* (M) and *SETD5* (Z, M). All five genes map to two distal adjacent TADs and are separated from rs6762644 by > 4 Mb. (b) 4q24-rs9790517 targets *CENPE* (Z, G) which maps to the boundary of a distal TAD and is separated from rs9790517 by ~ 2 Mb (c) 8q24.21-rs13281615 and rs11780156 target *CCDC26* (G, M). *CCDC26* maps close to the boundary of the TAD that lies adjacent to the capture region at ~ 1 Mb from rs11780156 and ~ 2Mb from rs13281615. (d) 11q13.1-rs3903072 and 11q13.3-rs554219, rs78540526 and rs75915166 target *CCND1* (all) and *FADD* (Br, M, Z). *CCND1* maps to a TAD that is distal to the 11q13.3 capture region, within the same TAD as the 11q13.1 capture region and *FADD* maps to a TAD that is distal to both capture regions. *CCND1* maps ~ 4Mb from rs3903072 and ~100 kb from the 11q13.3 SNPs; *FADD* maps ~ 4.5 Mb from rs3903072 and ~ 700 kb from the 11q13.3 SNPs.

Supplementary Figure 4: Box plots of *CCND1* expression according to rs3903072 genotype.

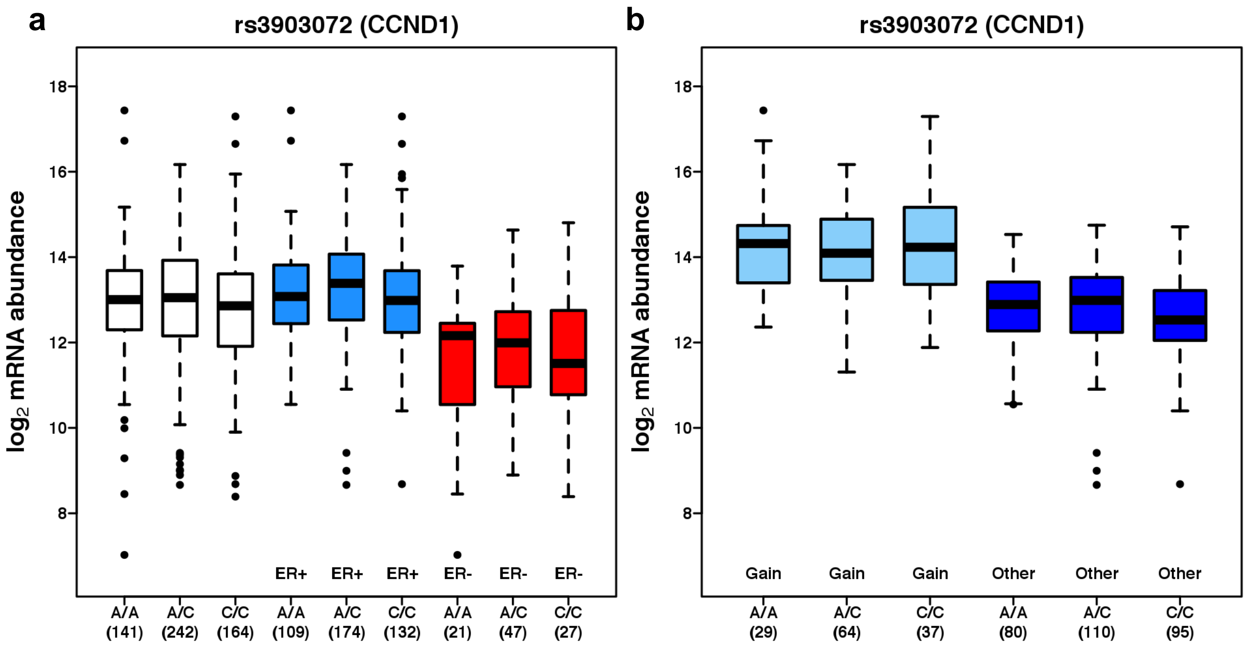

Legend: (a) Levels of expression of *CCND1* are associated with 11q13.1-rs3903072 genotype in all cancers (P = 0.03) and ER+ cancers (P = 0.04); (b) excluding samples with copy-number gains from the analysis of ER+ cancers did not alter the eQTL association (P = 0.05).

Supplementary Figure 5: Kaplan Meier plots of disease-specific survival according to levels of expression for *CENPE* (4q24), *TPCN2* (11q13.3), *ORAOV1* (11q13.3), *PDS5B* (13q13.1) and *SLC4A7* (3p24.1).

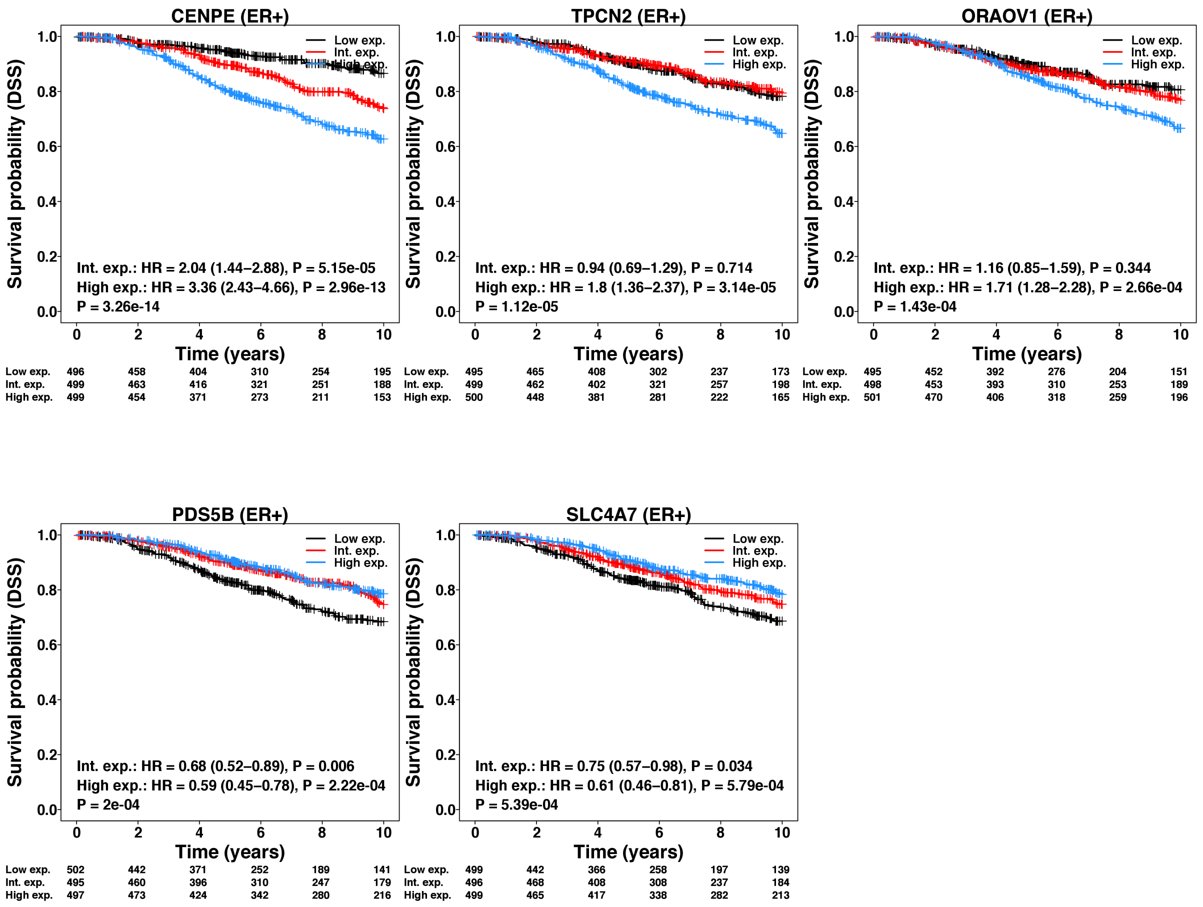

Legend: Levels of expression of *CENPE, TPCN2, ORAOV1, PDS5B, and SLC4A7* were all associated with disease-specific survival in ER+ cancers in the Metabric cohort (all FDR adjusted P < 0.005).

Supplementary Figure 6: Scatterplots showing the correlation between biological duplicates: both fragments captured.

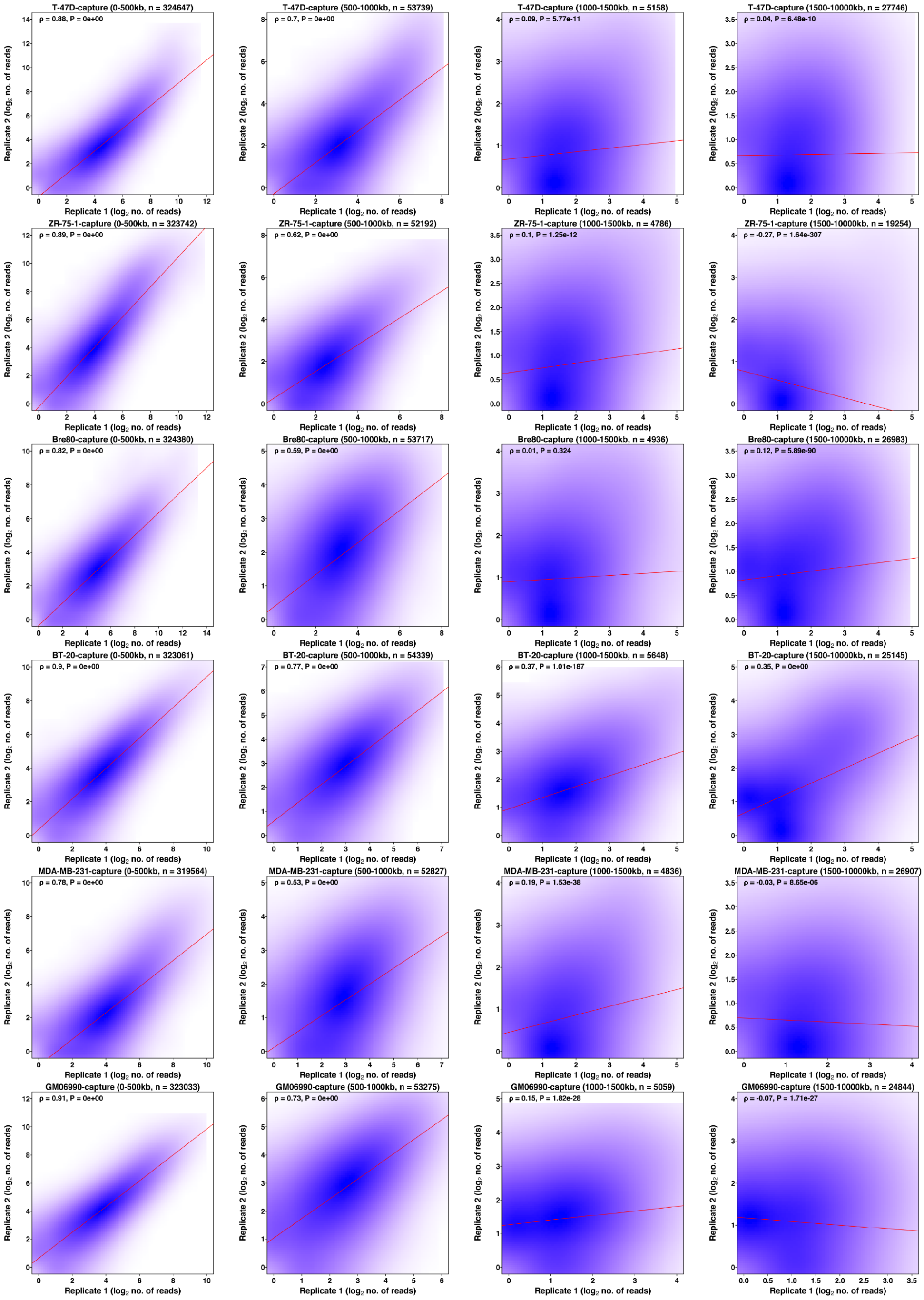

Legend: Scatterplot showing the association (quantified as Spearman's ρ) between duplicate libraries based on the number of raw di-tags mapping to each combination of captured HindIII fragments at each locus. The analysis was stratified by cell line and distance between interacting fragments. The intensity of blue colour indicates density of data points. The red line shows the linear regression fit.

Supplementary Figure 7: Scatterplots showing the correlation between biological duplicates: one fragment captured.

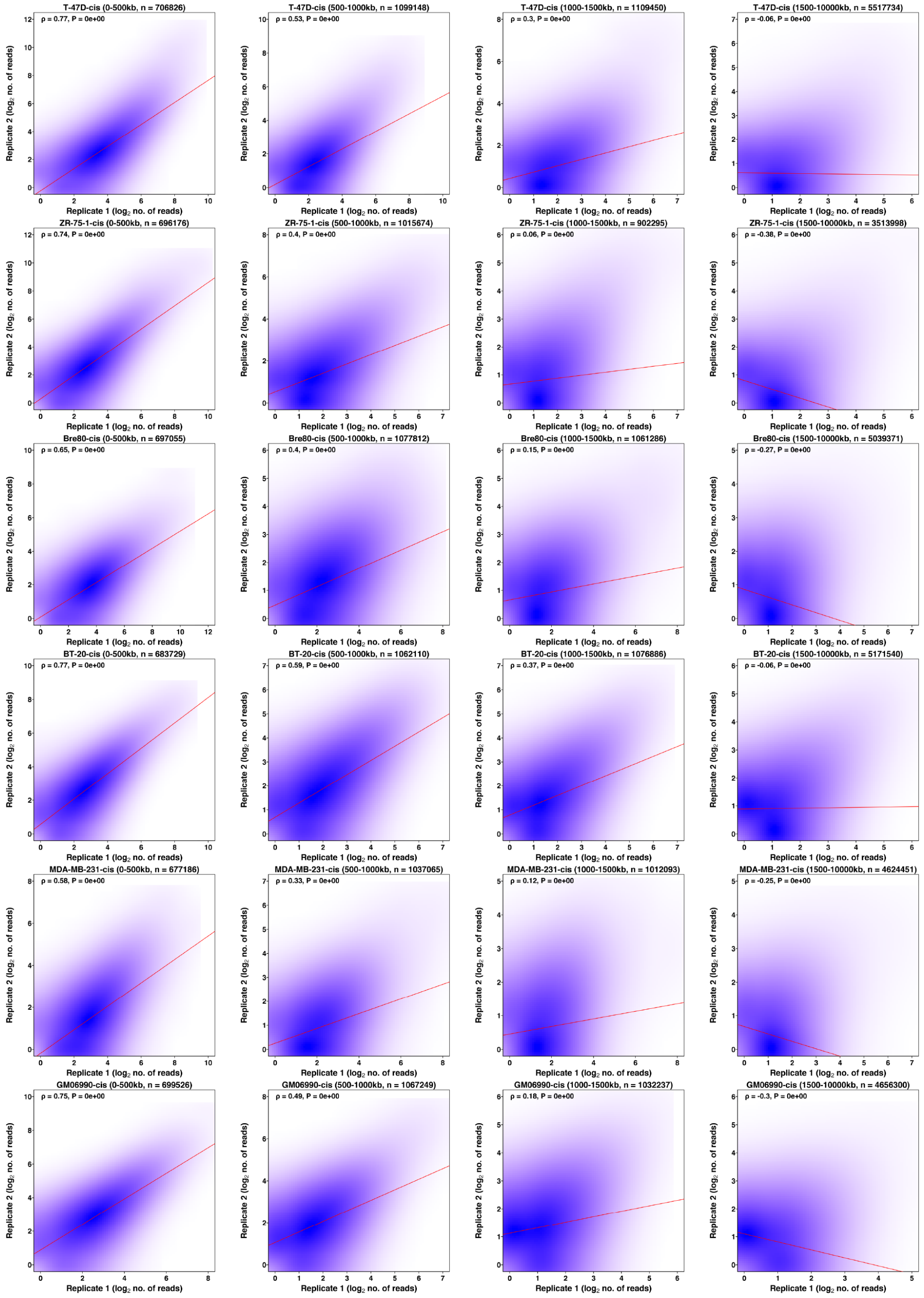

Legend: Scatterplot showing the association (quantified as Spearman's ρ) between duplicate libraries based on the number of raw di-tags mapping to each combination of captured HindIII and non-captured (cis) fragments at each locus. The analysis was stratified by cell line and distance between interacting fragments. The intensity of blue colour indicates density of data points. The red line shows the linear regression fit.

Supplementary Figure 8: Interaction peaks at 10q26.13 in T-47D and MDA-MB-231 cell lines, aligned with raw data.

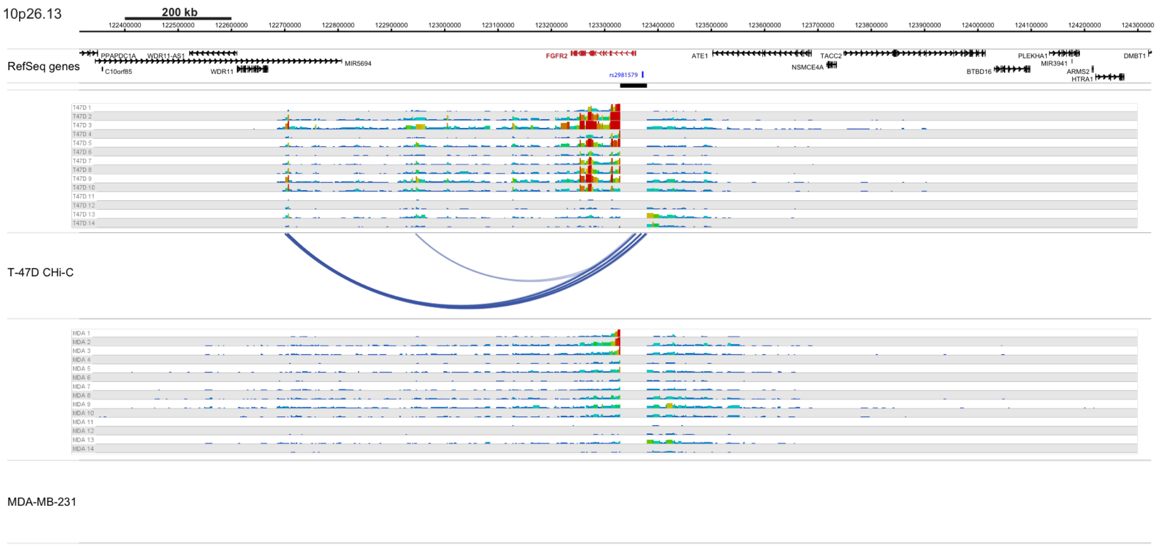

Legend: Interaction peaks, shown in a looping format, are aligned with raw data. Raw data is shown as one virtual 4C library per row (numbered T47D 1 -14 and MDA 1 – 14) such that each row shows the density of reads at each (non-captured) cis fragment forming di-tags with the single captured fragment. Interaction peaks between two captured fragments are red, interaction peaks between one captured fragment and one non-captured fragment are blue. Intensity of individual interactions are proportional to -$\log_2(P_{FDR})$. Capture regions are shown as black bars; data are aligned with genomic coordinates (hg19) and RefSeq genes. Target genes (ie the subset at which an interaction peak co-localises with the TSS) are shown in red. At this locus the majority of interaction peaks target consecutive HindIII fragments mapping to 122,701,138-122,708,722 bps (shown in detail in Fig 3b); interaction peaks are called for captured fragments T-47D 8-10 and 12 -14 (represented by this subset of rows). There are no statistically significant interaction peaks at this locus in MDA-MB-231.

Supplementary Figure 9: Interaction peaks at 11p15.5 in T-47D and MDA-MB-231 cell lines, aligned with raw data.

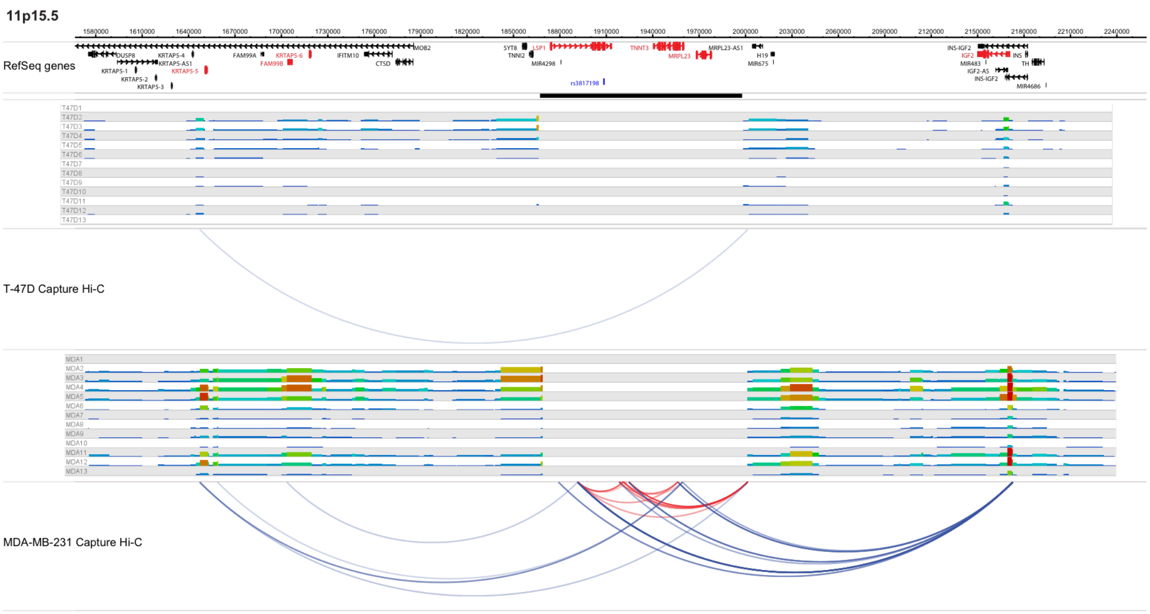

Legend: Interaction peaks and genomic features are as described in Supplementary Figure 8. At this locus, the predominant interaction peaks target uncaptured HindIII fragments mapping to 2169843 - 2173106 bps (colocalising with the *IGF2* TSS) and 1647384 – 1659371 bps (colocalising with the *KRTAP5-5* TSS) in MDA-MB-231. The peaks are specifically called for captured fragments MDA 2 – 5 and 11 – 13 (*IGF2*) and MDA 12 – 13 (*KRTAP5-5*) represented by this subset of rows). There are no significant interaction peaks targeting *IGF2* and just a single interaction peak targeting *KRTAP5-5* in T-47D.

Supplementary Tables

Supplementary Table 1: Numbers of interaction peaks according to the number of cell lines in which they were called (FDR corrected P < 0.01)

All loci		All loci excluding 8q21.11-rs2943559 and 8q24.21-rs13281615	
Number of Interaction peaks	Number of occurrences	Number of Interaction peaks	Number of occurrences
7,681 (60.3 %)	1	4,924 (50.6 %)	1
2,330 (18.3 %)	2	2,102 (21.6 %)	2
1,326 (10.4 %)	3	1,308 (13.4 %)	3
648 (5.1 %)	4	644 (6.6 %)	4
505 (4.0 %)	5	505 (5.2 %)	5
246 (1.9 %)	6	246 (2.5 %)	6

Supplementary Table 2: QC statistics for 12 CHi-C libraries

Library	Mapped di-tags	Valid di-tags	Valid (%)	Deduplicated di-tags	Deduplicated (%)	On target di-tags	On target (%)	Cis	Cis (%)	Trans	Trans (%)
T-47D (i)	153,068,398	118,827,997	77.6	98,388,392	82.8	71,192,391	72.4	39,259,451	55.1	31,932,940	44.9
T-47D (ii)	85,744,561	59,590,376	69.5	33,552,431	56.3	27,184,409	81.0	20,058,702	73.8	7,125,707	26.2
ZR-75-1 (i)	141,553,320	99,314,565	70.2	93,293,202	93.9	35,777,029	38.4	21,213,875	59.3	14,563,154	40.7
ZR-75-1 (ii)	97,359,853	47,839,452	49.1	37,163,213	77.7	23,770,004	64.0	19,177,229	80.7	4,592,775	19.3
BT-20 (i)	90,513,000	70,587,641	78.0	52,421,830	74.3	43,600,381	83.2	23,088,496	53.0	20,511,885	47.0
BT-20 (ii)	100,992,749	82,762,374	81.9	75,734,050	91.5	51,828,022	68.4	22,460,762	43.3	29,367,260	56.7
MDA-MB-231 (i)	98,958,940	75,682,339	76.5	57,566,138	76.1	47,914,248	83.2	23,520,035	49.1	24,394,213	50.9
MDA-MB-231 (ii)	97,867,931	48,810,657	49.9	42,194,379	86.4	32,906,011	78.0	9,670,919	29.4	23,235,092	70.6
Bre80 (i)	138,773,826	106,633,135	76.8	69,093,301	64.8	45,665,199	66.1	38,700,750	84.8	6,964,449	15.2
Bre80 (ii)	97,889,433	79,683,520	81.4	72,995,046	91.6	52,765,189	72.3	16,464,007	31.2	36,301,182	68.8
GM06990 (i)	119,162,182	76,656,519	64.3	74,218,586	96.8	29,601,946	39.9	17,915,075	60.5	11,686,871	39.5
GM06990 (ii)	102,319,005	78,596,635	76.8	58,687,568	74.7	48,340,624	82.4	24,788,802	51.3	23,551,822	48.7

These figures include di-tags mapping to 1,254 captured HindIII fragments that did not map to known breast cancer risk loci and were not considered further in this study.

Supplementary Table 3: ENCODE DNase I and ChIP-Seq data generated in T-47D cells (shown in Figure 2).

Marker/cell	Data type	Source	Laboratory	GEO accession
T-47D				
DNase (DMSO)	peaks	ENCODE/OpenChrom	Duke	GSM816673
DNase (E_2)	peaks	ENCODE/OpenChrom	Duke	GSM1008576
CTCF (DMSO)	peaks	ENCODE	Myers/Hudson-Alpha	GSM803348
FOXA1 (DMSO)	peaks	ENCODE	Myers/Hudson-Alpha	GSM803409
GATA3 (DMSO)	peaks	ENCODE	Myers/Hudson-Alpha	GSM803514
p300 (DMSO)	peaks	ENCODE	Myers/Hudson-Alpha	GSM803522
ERα (E_2)	peaks	ENCODE	Myers/Hudson-Alpha	GSM803539
ERα (Genistein)	peaks	ENCODE	Myers/Hudson-Alpha	GSM803374
