# Supplementary material for: Capture Hi-C identifies putative target genes at 33 breast cancer risk loci
Source: Nat Commun. 2018 Mar 12;9:1028. doi: 10.1038/s41467-018-03411-9 (PMC5847529; doi:10.1038/s41467-018-03411-9)
Supplement: Supplementary file 3 — Description of Additional Supplementary Files [file 41467_2018_3411_MOESM3_ESM.pdf]

Description of Additional Supplementary Files

File Name: Supplementary Data 1
Description: Capture regions (defined as the region that included all SNPs that were correlated ($r2 \geq 0.2$) with the published SNP based on 1000 Genomes pilot data) for 63 GWAS risk loci that were included in this study.

File Name: Supplementary Data 2
Description: The number of statistically significant interaction peaks at each locus, in each cell line. A statistically significant interaction peak was defined as any pair of HindIII fragments for which the number of di-tags was significantly (FDR adjusted $P < 0.01$) greater than expected under a negative binomial model, taking into account both the distance between the HindIII fragments and the propensity of the bait fragment to form interactions (''interactability'').

File Name: Supplementary Data 3
Description: The subset of interaction peaks that were present in at least two cell lines are provided in a format that can be uploaded onto the WashU browser.
Columns are:
Coordinates of interacting fragment 1
Coordinates of interacting fragment 2
$-\log_2$ of the FDR corrected P-value for this interaction peak
Cell line in which the interaction peak was observed
Total number of cell lines in which this particular interaction peak was observed (by definition this is 2 – 6)

File Name: Supplementary Data 4
Description: Risk loci which formed interaction peaks with target genes and the cell lines in which these interaction peaks were detected: T-47D (T), ZR-75-1 (Z), Bre80 (Br), BT-20 (BT), MDA-MB-231 (M) and GM06990 (G).

File Name: Supplementary Data 5
Description: Distances between published risk SNPs and putative CHi-C target genes (kb) at 36 informative risk loci. Distances are given in parenthesis after the gene name.

File Name: Supplementary Data 6
Description: eQTL analysis of 69 protein coding target genes at 26 risk loci in TCGA breast cancer data. Loci at which there was no suitable genotyped tag SNP (defined as a SNP that was correlated with the published risk SNP with $r2 \geq 0.8$) were excluded.

File Name: Supplementary Data 7
Description: Disease-specific survival analysis of 97 target genes in Metabric data based on all cancers (N = 1970), ER+ cancers (N = 1494) and ERcancers (N = 433).
