# Supplementary material for: Capture Hi-C identifies putative target genes at 33 breast cancer risk loci
Source: Nat Commun. 2018 Mar 12;9:1028. doi: 10.1038/s41467-018-03411-9 (PMC5847529; doi:10.1038/s41467-018-03411-9)
Supplement: Supplementary file 4 — Supplementary Data 1 [file 41467_2018_3411_MOESM4_ESM.docx]

**Supplementary Data 1: Captured genomic regions**

| Locus | SNP | Start coordinates | End coordinates | Size  (bps) | HindIII fragments | Reference |
| --- | --- | --- | --- | --- | --- | --- |
| 1p36.22 | rs616488 | 10,259,917 | 10,638,604 | 378,687 | 102 | [Michailidou et al., 2013](#_ENREF_38) |
| 1p13.2 | rs11552449 | 114,075,796 | 114,525,636 | 449,840 | 139 | [Michailidou et al., 2013](#_ENREF_38) |
| 1p11.2 | rs11249433 | 120,804,559 | 121,482,515 | 677,956 | 204 | [Thomas et al., 2009](#_ENREF_49) |
| 2p24.1 | rs12710696 | 19,302,429 | 19,461,524 | 159,095 | 43 | [Garcia-Closas et al., 2013](#_ENREF_20) |
| 2q14.2 | rs4849887 | 121,226,256 | 121,264,471 | 38,215 | 7 | [Michailidou et al., 2013](#_ENREF_38) |
| **2q31.1** | rs2016394 | 172,953,438 | 172,974,566 | 21,128 | 6 | [Michailidou et al., 2013](#_ENREF_38) |
| **2q31.1** | rs1550623 | 174,198,854 | 174,248,583 | 49,729 | 12 | [Michailidou et al., 2013](#_ENREF_38) |
| **2q35** | rs13387042 | 217,856,966 | 217,941,790 | 84,824 | 23 | [Stacey et al., 2007](#_ENREF_47) |
| **2q35** | rs16857609 | 218,239,239 | 218,394,530 | 155,291 | 49 | [Michailidou et al., 2013](#_ENREF_38) |
| 3p26.1 | rs6762644 | 4,727,915 | 4,789,051 | 61,136 | 26 | [Michailidou et al., 2013](#_ENREF_38) |
| **3p24.1** | rs4973768 | 27,038,340 | 27,552,361 | 514,021 | 154 | [Ahmed et al., 2009](#_ENREF_1) |
| **3p24.1** | rs12493607 | 30,664,321 | 30,689,755 | 25,434 | 6 | [Michailidou et al., 2013](#_ENREF_38) |
| 4q24 | rs9790517 | 106,024,327 | 106,448,002 | 423,675 | 137 | [Michailidou et al., 2013](#_ENREF_38) |
| 4q34.1 | rs6828523 | 175,740,771 | 175,925,281 | 184,510 | 63 | [Michailidou et al., 2013](#_ENREF_38) |
| 5p15.33 | rs10069690, rs7726159, rs2736108 | 1,270,983 | 1,325,590 | 54,607 | 10 | [Haiman et al., 2011](#_ENREF_26);  [Bojesen et al., 2013](#_ENREF_6) |
| 5p12 | rs10941679 | 44,425,291 | 45,074,633 | 649,342 | 231 | [Stacey et al., 2008](#_ENREF_50);  [Ghoussaini et al., 2016](#_ENREF_23) |
| **5q11.2** | rs889312 | 55,990,342 | 56,276,198 | 285,856 | 95 | [Easton et al., 2007](#_ENREF_13) |
| **5q11.2** | rs1353747, rs10472076 | 58,177,646 | 58,407,778 | 230,132 | 78 | [Michailidou et al., 2013](#_ENREF_38) |
| 5q33.3 | rs1432679 | 158,165,641 | 158,381,663 | 216,022 | 76 | [Michailidou et al., 2013](#_ENREF_38) |
| 6p25.3 | rs11242675 | 1,305,730 | 1,347,198 | 41,468 | 9 | [Michailidou et al., 2013](#_ENREF_38) |
| 6p23 | rs204247 | 13,628,357 | 13,751,547 | 123,190 | 40 | [Michailidou et al., 2013](#_ENREF_38) |
| 6q14.1 | rs17529111 | 81,961,349 | 82,390,104 | 428,755 | 136 | [Siddiq et al., 2012](#_ENREF_47) |
| 6q25.1 | rs12662670, rs2046210 | 151,844,071 | 152,020,682 | 161,484 | 56 | [Zheng et al., 2009](#_ENREF_54);  [Hein et al., 2012](#_ENREF_27) |
| 7q35 | rs720475 | 144,019,775 | 144,174,291 | 154,516 | 41 | [Michailidou et al., 2013](#_ENREF_38) |
| 8p12 | rs9693444 | 29,417,238 | 29,537,217 | 119,979 | 43 | [Michailidou et al., 2013](#_ENREF_38) |
| **8q21.11** | rs6472903 | 76,113,426 | 76,260,998 | 147,572 | 48 | [Michailidou et al., 2013](#_ENREF_38) |
| **8q21.11** | rs2943559 | 76,295,292 | 76,700,618 | 405,326 | 125 | [Michailidou et al., 2013](#_ENREF_38) |
| **8q24.21** | rs13281615 | 128,313,360 | 128,400,176 | 86,816 | 21 | [Easton et al., 2007](#_ENREF_13) |
| **8q24.21** | rs11780156 | 129,119,667 | 129,275,391 | 155,724 | 56 | [Michailidou et al., 2013](#_ENREF_38) |
| 9p21.3 | rs1011970 | 21,951,902 | 22,121,357 | 169,455 | 47 | [Turnbull et al., 2010](#_ENREF_50) |
| **9q31.2** | rs10759243 | 110,277,340 | 110,331,404 | 54,064 | 10 | [Michailidou et al., 2013](#_ENREF_38) |
| **9q31.2** | rs865686 | 110,881,453 | 111,073,347 | 191,894 | 48 | [Fletcher et al., 2011](#_ENREF_17) |
| 10p15.1 | rs2380205 | 5,753,516 | 5,982,280 | 228,764 | 60 | [Turnbull et al., 2010](#_ENREF_50) |
| 10p12.31 | rs11814448, rs7072776 | 21,667,042 | 22,729,537 | 1,062,495 | 343 | [Michailidou et al., 2013](#_ENREF_38) |
| 10q21.2 | rs10995190 | 64,268,659 | 64,304,197 | 35,538 | 9 | [Turnbull et al., 2010](#_ENREF_50) |
| 10q22.3 | rs704010 | 80,809,803 | 80,859,755 | 49,952 | 10 | [Turnbull et al., 2010](#_ENREF_50) |
| 10q25.2 | rs7904519 | 114,722,134 | 114,823,426 | 101,292 | 41 | [Michailidou et al., 2013](#_ENREF_38) |
| 10q26.13 | rs2981579 | 123,329,667 | 123,379,947 | 50,280 | 14 | [Easton et al., 2007](#_ENREF_13) |
| 11p15.5 | rs3817198 | 1,876,594 | 1,960,119 | 83,525 | 13 | [Easton et al., 2007](#_ENREF_13) |
| **11q13.1** | rs3903072 | 65,412,467 | 65,782,768 | 370,301 | 49 | [Michailidou et al., 2013](#_ENREF_38) |
| **11q13.3** | rs554219, rs78540526, rs75915166 | 69,283,007 | 69,509,669 | 226,662 | 48 | [Turnbull et al., 2010](#_ENREF_50); [French et al., 2013](#_ENREF_19) |
| 11q24.3 | rs11820646 | 129,452,082 | 129,480,334 | 28,252 | 10 | [Michailidou et al., 2013](#_ENREF_38) |
| 12p13.1 | rs12422552 | 14,354,783 | 14,427,126 | 72,343 | 15 | [Michailidou et al., 2013](#_ENREF_38) |
| 12p11.22 | rs10771399 | 27,975,352 | 28,579,324 | 603,972 | 194 | [Ghoussaini et al., 2012](#_ENREF_22) |
| 12q22 | rs17356907 | 96,020,785 | 96,037,389 | 16,604 | 6 | [Michailidou et al., 2013](#_ENREF_38) |
| 12q24.21 | rs1292011 | 115,813,607 | 115,838,648 | 25,041 | 7 | [Ghoussaini et al., 2012](#_ENREF_22) |
| 13q13.1 | rs11571833 | 32,920,321 | 33,487,779 | 567,458 | 186 | [Michailidou et al., 2013](#_ENREF_38) |
| 14q13.3 | rs2236007 | 37,030,096 | 37,136,545 | 106,449 | 33 | [Michailidou et al., 2013](#_ENREF_38) |
| **14q24.1** | rs2588809 | 68,103,993 | 68,813,115 | 709,122 | 216 | [Orr et al., 2012](#_ENREF_44) |
| **14q24.1** | rs999737 | 68,877,529 | 69,069,416 | 191,887 | 55 | [Thomas et al., 2009](#_ENREF_49) |
| 14q32.11 | rs941764 | 91,818,025 | 91,971,886 | 153,861 | 51 | [Michailidou et al., 2013](#_ENREF_38) |
| **16q12.1** | rs3803662 | 52,535,810 | 52,646,376 | 110,566 | 34 | [Stacey et al., 2007](#_ENREF_47) |
| **16q12.2** | rs17817449, rs11075995 | 53,797,565 | 53,877,592 | 80,027 | 22 | [Easton et al., 2007](#_ENREF_13); [Garcia-Closas et al., 2013](#_ENREF_20) |
| 16q23.2 | rs13329835 | 80,607,000 | 80,676,117 | 69,117 | 17 | [Michailidou et al., 2013](#_ENREF_38) |
| 17q22 | rs6504950 | 52,974,643 | 53,271,918 | 297,275 | 93 | [Ahmed et al., 2009](#_ENREF_1) |
| **18q11.2** | rs527616 | 24,305,145 | 24,339,719 | 34,574 | 8 | [Michailidou et al., 2013](#_ENREF_38) |
| **18q11.2** | rs1436904 | 24,481,272 | 24,625,756 | 144,484 | 53 | [Michailidou et al., 2013](#_ENREF_38) |
| **19p13.11** | rs8170, rs2363956 | 17,311,308 | 17,451,187 | 139,879 | 14 | [Antoniou et al., 2010](#_ENREF_2) |
| **19p13.11** | rs4808801 | 18,510,767 | 18,652,844 | 142,077 | 19 | [Michailidou et al., 2013](#_ENREF_38) |
| 19q13.31 | rs3760982 | 44,281,094 | 44,433,904 | 152,810 | 45 | [Michailidou et al., 2013](#_ENREF_38) |
| 21q21.1 | rs2823093 | 16,460,759 | 16,575,042 | 114,283 | 36 | [Ghoussaini et al., 2012](#_ENREF_22) |
| 22q12.1 | rs17879961, rs132390 | 28,739,115 | 29,977,318 | 1,238,203 | 313 | [Michailidou et al., 2013](#_ENREF_38) |
| 22q13.1 | rs6001930 | 40,469,249 | 41,062,014 | 592,765 | 162 | [Michailidou et al., 2013](#_ENREF_38) |
| Total |  |  |  | 14,699,601 | 4,317 |  |

5p15.33-rs10069690, rs7726159 and rs2736108, 11q13.3-rs554219, rs78540526 and rs75915166, 16q12.2-rs17817449 and rs11075995, 19p13.1-rs8170 and rs2363956 were originally designed as eight separate capture regions (Methods) but were collapsed into four regions due to the proximity of the published risk SNPs. One region 10q23.1-rs7071985 (82909977-83064943) failed to generate high numbers of reads in any of the cell lines we assayed and was excluded from the analysis. Pairs of adjacent risk loci that map within 5Mb of each other are indicated in bold; there were significant interaction peaks between adjacent risk loci for all pairs except those at 5q11.2, 8q24.21, 16q12.1-2 and 18q11.2.
