# Supplementary material for: Capture Hi-C identifies putative target genes at 33 breast cancer risk loci
Source: Nat Commun. 2018 Mar 12;9:1028. doi: 10.1038/s41467-018-03411-9 (PMC5847529; doi:10.1038/s41467-018-03411-9)
Supplement: Supplementary file 5 — Supplementary Data 2 [file 41467_2018_3411_MOESM5_ESM.docx]

**Supplementary Data 2: Numbers of statistically significant interaction peaks in six cell lines at 51 informative loci and 12 uninformative loci**

| **Locus** | **SNP** | **T-47D** | **ZR-75-1** | **Bre80** | **BT-20** | **MDA-MB-231** | **GM06990** |
| --- | --- | --- | --- | --- | --- | --- | --- |
| **Informative loci** |  |  |  |  |  |  |  |
| 1p36.22 | rs616488 | 3 | 0 | 45 | 1 | 16 | 1 |
| 1p13.2 | rs11552449 | 2 | 2 | 5 | 3 | 0 | 0 |
| 1p11.2 | rs11249433 | 0 | 307 | 0 | 0 | 0 | 0 |
| 2p24.1 | rs12710696 | 15 | 3 | 0 | 0 | 0 | 0 |
| 2q31.1 | rs2016394 | 36 | 24 | 57 | 243 | 5 | 17 |
| 2q31.1 | rs1550623 | 0 | 0 | 1 | 0 | 0 | 2 |
| 2q35 | rs13387042 | 42 | 56 | 0 | 21 | 4 | 0 |
| 2q35 | rs16857609 | 323 | 335 | 19 | 112 | 13 | 10 |
| 3p26.1 | rs6762644 | 522 | 220 | 181 | 147 | 161 | 140 |
| 3p24.1 | rs4973768 | 104 | 85 | 52 | 0 | 0 | 0 |
| 3p24.1 | rs12493607 | 161 | 0 | 19 | 0 | 0 | 0 |
| 4q24 | rs9790517 | 11 | 10 | 14 | 0 | 1 | 155 |
| 5p15.33 | rs10069690, rs7726159, rs2736108 | 0 | 0 | 0 | 18 | 1 | 0 |
| 5p12 | rs10941679 | 0 | 90 | 21 | 0 | 0 | 0 |
| 5q11.2 | rs889312 | 101 | 49 | 53 | 0 | 8 | 2 |
| 5q11.2 | rs1353747, rs10472076 | 0 | 1 | 7 | 0 | 0 | 0 |
| 5q33.3 | rs1432679 | 22 | 31 | 0 | 0 | 0 | 0 |
| 6p25.3 | rs11242675 | 4 | 0 | 2 | 0 | 0 | 0 |
| 6p23 | rs204247 | 0 | 0 | 5 | 0 | 8 | 0 |
| 6q25.1 | rs12662670, rs2046210 | 32 | 9 | 0 | 0 | 0 | 1 |
| 7q35 | rs720475 | 0 | 0 | 3 | 0 | 0 | 0 |
| 8p12 | rs9693444 | 0 | 0 | 38 | 0 | 13 | 0 |
| 8q21.11 | rs6472903 | 0 | 4 | 0 | 0 | 0 | 0 |
| 8q21.11 | rs2943559 | 114 | 1744 | 11 | 0 | 0 | 33 |
| 8q24.21 | rs13281615 | 1007 | 4 | 4 | 0 | 5 | 85 |
| 8q24.21 | rs11780156 | 17 | 48 | 76 | 0 | 103 | 6 |
| 9p21.3 | rs1011970 | 0 | 56 | 68 | 0 | 0 | 11 |
| 9q31.2 | rs10759243 | 27 | 11 | 24 | 3 | 18 | 0 |
| 9q31.2 | rs865686 | 26 | 38 | 11 | 3 | 3 | 0 |
| 10p12.31 | rs7072776, rs11814448 | 6 | 11 | 67 | 10 | 50 | 51 |
| 10q22.3 | rs704010 | 7 | 4 | 9 | 0 | 32 | 0 |
| 10q26.13 | rs2981579 | 15 | 14 | 14 | 0 | 0 | 0 |
| 11p15.5 | rs3817198 | 1 | 0 | 0 | 199 | 28 | 0 |
| 11q13.1 | rs3903072 | 220 | 274 | 118 | 246 | 466 | 99 |
| 11q13.3 | rs554219, rs78540526 | 319 | 26 | 91 | 100 | 344 | 43 |
| 12p13.1 | rs12422552 | 0 | 8 | 23 | 0 | 4 | 12 |
| 12p11.22 | rs10771399 | 0 | 1 | 15 | 0 | 1 | 0 |
| 12q24.21 | rs1292011 | 3 | 1 | 8 | 3 | 0 | 0 |
| 13q13.1 | rs11571833 | 14 | 0 | 3 | 32 | 0 | 17 |
| 14q13.3 | rs2236007 | 50 | 61 | 64 | 0 | 0 | 0 |
| 14q24.1 | rs2588809 | 240 | 68 | 51 | 26 | 33 | 114 |
| 14q24.1 | rs999737 | 0 | 1 | 0 | 15 | 0 | 0 |
| 16q12.2 | rs17817449, rs11075995 | 298 | 229 | 10 | 11 | 18 | 0 |
| 16q23.2 | rs13329835 | 82 | 7 | 0 | 0 | 0 | 0 |
| 17q22 | rs6504950 | 231 | 30 | 63 | 0 | 1 | 1 |
| 18q11.2 | rs1436904 | 0 | 24 | 3 | 0 | 0 | 0 |
| 19p13.11 | rs8170, rs2363956 | 0 | 0 | 2 | 84 | 3 | 3 |
| 19p13.11 | rs4808801 | 2 | 0 | 8 | 44 | 14 | 3 |
| 19q13.31 | rs3760982 | 0 | 0 | 1 | 0 | 0 | 2 |
| 22q12.1 | rs17879961, rs132390 | 0 | 2 | 5 | 0 | 0 | 0 |
| 22q13.1 | rs6001930 | 5 | 12 | 11 | 1 | 4 | 9 |
| **Uninformative loci** | |  |  |  |  |  |  |
| 2q14.2 | rs4849887 | 0 | 0 | 0 | 0 | 0 | 0 |
| 4q34.1 | rs6828523 | 0 | 0 | 0 | 0 | 0 | 0 |
| 6q14.1 | rs17529111 | 0 | 0 | 0 | 0 | 0 | 0 |
| 10p15.1 | rs2380205 | 0 | 0 | 0 | 0 | 0 | 0 |
| 10q21.2 | rs10995190 | 0 | 0 | 0 | 0 | 0 | 0 |
| 10q25.2 | rs7904519 | 0 | 0 | 0 | 0 | 0 | 0 |
| 11q24.3 | rs11820646 | 0 | 0 | 0 | 0 | 0 | 0 |
| 12q22 | rs17356907 | 0 | 0 | 0 | 0 | 0 | 0 |
| 14q32.11 | rs941764 | 0 | 0 | 0 | 0 | 0 | 0 |
| 16q12.1 | rs3803662 | 0 | 0 | 0 | 0 | 0 | 0 |
| 18q11.2 | rs527616 | 0 | 0 | 0 | 0 | 0 | 0 |
| 21q21.1 | rs2823093 | 0 | 0 | 0 | 0 | 0 | 0 |
